# Optimization of athletic pasta formulation by D‐optimal mixture design

**DOI:** 10.1002/fsn3.1764

**Published:** 2020-07-08

**Authors:** Leila Kamali Rousta, Amir Pouya Ghandehari Yazdi, Mahdi Amini

**Affiliations:** ^1^ Department of Food Research and Development Zar Research and Industrial Development Group Alborz Iran

**Keywords:** athletic, D‐optimal, fortification, optimization, pasta

## Abstract

The aim of this study was to produce an athletic pasta by the addition of various sources of protein. For this purpose, D‐optimal mixture design used for optimization of formulation of athletic pasta and protein with considering the hardness as main parameter. Various properties of the optimized formulation were evaluated. The optimal formulation contained 45.41% of semolina, 24% of pea protein isolate (PPI), 18% of oat flour (OF), 5% of soy protein isolate (SPI), 5% whey protein isolate (WPI), and 2% of gluten (G). In optimized formulation, the protein content increased by more than 2.9 times compared to control with the hardness in the range (569 g). Hardness, optimal cooking time, and cooking loss of products increased as the level of protein increased. The optimal formulation had a higher sensory acceptance than the control, which is probably related to color changes. Due to the amount and biological value of the proteins used and the high acceptance obtained, this formulation can be suggested for athletes. The obtained results indicated that production of athletic pasta with high biological value by using mixture of SPI, PPI, WPI, OF, and G is possible.

## Graphical abstract

Athletic pasta formulation was optimized by D‐optimal mixture design approach. Production of pasta with high protein content and suitable for athletes. Production of pasta with higher overall acceptability compared to the commercial sample.

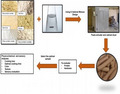

## 1 INTRODUCTION

Pasta is one of the most popular foods consumed in the world because of its low price, easy cooking, good taste, nutritional attribute, palatability, and low glycemic index. Pasta is commonly made from durum wheat semolina, which is rich in carbohydrate, but it is poor in dietary fibers, minerals, proteins, and vitamins (Armellini et al., 2019). Also, wheat semolina proteins are deficient in lysine, methionine, and essential amino acids (Alireza Sadeghi & Bhagya, 2008). So far, several studies have been done to increase the protein content of pasta (Desai, Brennan, & Brennan, 2018; Kumar et al., 2019; Teterycz, Sobota, Zarzycki, & Latoch, 2020). Legumes such as pea contain high level of proteins, and essential amino acids such as lysine while the amount of sulfur‐containing amino acids in legume proteins are low. Therefore, by combining cereals with legumes, a complete profile of proteins in pasta can be achieved (Duranti, 2006). Various legumes such as peanut (Howard & Hung, 2010), faba (Laleg et al., 2017), chickpea (Kore, 2018; Shyam, Mishra, Vaidya, & Sharma, 2017), and soybean (Marengo et al., 2018) have been used in numerous studies to improve the nutritional value of pasta.

Another way to enrich pasta with protein is to use milk proteins. Whey is one of the milk proteins that is a good source of essential amino acids. Whey has been used in many studies to enrich various foods such vermicelli (Prabhasankar, Rajiv, Indrani, & Rao, 2007), noodles (Baskaran et al., 2011), and pasta from sweet potato (Gopalakrishnan, Menon, Padmaja, Sajeev, & Moorthy, 2011).

According to European Union legislations, high protein products such as high protein‐enriched pasta can be declared as a source of protein and high protein content, while the amount of energy derived from the protein in that food is at least 12 and 20 percent, respectively (Gilsenan, 2011). Foods rich in protein increase satiety compared to foods rich in fat or carbohydrates (Vozzo et al., 2003).

In this study, the combination of isolated soy protein (90%), isolated whey protein (80%), isolated pea protein (80%), oat flour, and gluten powder was used to enrich the pasta. Oat flour is a great source of dietary fiber, especially beta glucan that reduces the risk of diabetes, cardiovascular disease, blood cholesterol, and obesity. Oat has a higher protein than the other grains, as well as is a good source of vitamins, minerals, and antioxidants (Spiller, 2001). Enrichment with components is a good way to improve the nutritional properties of pasta, but it may have a negative effect on their texture properties.

This study was conducted to evaluate the (a) possibility of producing a new functional pasta by increased protein and fiber and (b) the physicochemical properties, nutritional, and sensory attributes of enriched pasta. To the best of our knowledge, this is the first study in the literature focused on production of pasta designed for athletes by the combination of soy protein isolate, whey protein isolate, pea protein isolate, oat flour, and gluten powder.

## 2 MATERIALS AND METHODS

### 2.1 Materials

Raw materials including semolina, pea protein isolate, whey protein isolate, soy protein isolate, oat flour, and gluten were purchased from Zar Semolina Co, Roquette Co, Hilmar Co, Ardineh Co, and CFF GmbH and Co. KG, respectively. All chemicals were purchased from Sigma‐Aldrich Srl.

### 2.2 Experimental design

Design‐Expert 7.1.5 (Stat‐Ease Inc.) software was used to define the optimum proportions of the enriched pasta formulation. In this study, D‐optimal design was used with six components: semolina (S), pea protein isolate 80% (PPI), whey protein isolate 80% (WPI), soy protein isolate 90% (SPI), oat flour (OF), and gluten (G). Table 1 displays the composition of each blend calculated from the experimental design. The amount of the components was selected based on preliminary tests (S: 30%–60%, PPI: 5%–30%, WPI: 5%–10%, SPI: 5%–12%, OF: 7–18, G: 2%–5%). Design‐Expert software designed 31 samples (Table 1). Effects of semolina, pea protein isolate, whey protein isolate, soy protein isolate, oat flour, and gluten on the attributes of pasta were investigated, and optimum mixture was selected. After selecting the optimal sample (OS) based on protein and texture, its physicochemical properties, nutritional value, and sensory attributes were compared with the control sample.

**TABLE 1:** Experimental design showing the doses of the components used in the formulation

RUN	S	SPI	PPI	WPI	OF	G
1	60.00	12.00	5.00	7.50	13.50	2.00
2	47.50	5.00	17.50	10.00	18.00	2.00
3	30.00	11.00	30.00	9.00	18.00	2.00
4	31.00	6.00	30.00	10.00	18.00	5.00
5	60.00	6.00	6.00	5.00	18.00	5.00
6	44.00	12.00	30.00	5.00	7.00	2.00
7	40.00	5.00	30.00	5.00	18.00	2.00
8	30.00	12.00	30.00	5.00	18.00	5.00
9	36.00	12.00	30.00	10.00	7.00	5.00
10	60.00	12.00	9.00	10.00	7.00	2.00
11	60.00	5.00	13.00	10.00	7.00	5.00
12	46.00	5.00	30.00	10.00	7.00	2.00
13	60.00	12.00	11.00	5.00	7.00	5.00
14	60.00	8.50	5.00	10.00	14.50	2.00
15	48.00	5.00	30.00	5.00	7.00	5.00
16	60.00	10.00	5.00	5.00	18.00	2.00
17	60.00	5.00	5.00	8.50	18.00	3.50
18	45.00	8.50	17.50	7.50	18.00	3.50
19	60.00	5.00	21.00	5.00	7.00	2.00
20	30.00	12.00	30.00	10.00	14.50	3.50
21	50.00	12.00	5.00	10.00	18.00	5.00
22	49.50	12.00	19.50	10.00	7.00	2.00
23	39.20	10.44	24.08	8.83	13.91	3.51
24	54.20	8.19	12.83	6.33	15.66	2.76
25	46.95	6.94	24.08	6.33	12.91	2.76
26	53.00	5.00	23.00	10.00	7.00	2.00
27	48.00	5.00	30.00	5.00	7.00	5.00
28	36.00	12.00	30.00	10.00	7.00	5.00
29	31.00	6.00	30.00	10.00	18.00	5.00
30	30.00	12.00	30.00	5.00	18.00	5.00
31	50.00	12.00	5.00	10.00	18.00	5.00

Abbreviations: G, gluten; OF, oat flour; PPI, pea protein isolate 80%; S, Semolina; SPI, soy protein isolate 90%; WPI, whey protein isolate 80%.

### 2.3 Pasta preparation

Preparation of pasta was done according to the formulation of Armellini et al. (2019). Control sample formula consisted of 165 g semolina and 70 ml water. For preparation of pasta, semolina and water were mixed continuously for 10 min in a chamber of pasta extruder (Anselmo, Bene Vagienna, Italy). The mixture was extruded at the 25°C and dried in the cabinet dryer (Anselmo Bene Vagienna, Italy) at 75 ± 2°C for 5 hr to achieve the moisture content of 8%–9%. According to Table 1, enriched samples were prepared by the method described above.

### 2.4 Chemical analysis

The chemical compositions of raw materials and pasta products (Control and OS) were determined according to the AACC method (Association of Official Agricultural Chemists, 2000). Results reported in g/100 g (Dry basis).

### 2.5 Cooking properties of samples

Cooking loss and optimal cooking time (OCT) were evaluated according to the methods of described by Tudorica, Kuri, and Brennan. (2002).

### 2.6 Color analysis

Hunter ColorFlex colorimeter (Hunter Lab was used to evaluate the color of samples by L*(black (0) to white (100)), a* (red to green), and b* (yellow to blue) values (Ghandehari Yazdi, Barzegar, Ahmadi Gavlighi, Sahari, & Mohammadian, 2020.

### 2.7 Textural analysis

Hardness (maximum force during the first compression) and adhesiveness (negative area after the first compression) of cooked samples (in OCT) were evaluated using a TA‐XT plus texture analyzer (Stable Micro System), equipped with a stainless steel cylindrical probe (diameter: 75 mm). The conditions of analysis were as follows: load cell = 10 kg, test speed, and post‐test speed: 1 mm/s; distance: 50 mm in compression mode; time: 1 s. Contact force = 5 g. Hardness was defined as the maximum compression force (g) during the testing process of the sample (Petitot, Boyer, Minier, & Micard, 2010).

### 2.8 Sensory analysis

Sensory analysis of the uncooked and cooked samples was done to evaluate the flavor, color, texture, and overall liking. Samples were investigated by 50 semitrained panelists (25 male, 25 female, age range 20–35 years old) that they were selected from the athletes. For this purpose, 50 g of samples (control or OS) was cooked at OCT in 250 ml boiling water. Panelists were asked to provide liking scores from 1 to 9 (1: extremely unpleasant, to 9: extremely pleasant) for each attributes of samples (Biró, Fodor, Szedljak, Pásztor‐Huszár, & Gere, 2019).

### 2.9 Statistical analysis

Linear, quadratic, and special cubic models were evaluated (Equation 2, 3, and 4, respectively), and these models were fitted to each of the responses (hardness, protein) with the independent variables. All tests were done in triplicates, and the mean ± standard deviation of the data was reported. Least significant difference (LSD) test was used to identify the presence of significant differences at 95% confidence level. For this aim, statistical analysis was accomplished using SPSS software (version 22, SPSS Inc).

Y = b1A+b2B + b3C+b4D + b5E+b6F (linear, 2)

Y = b1A+b2B + b3C+b4D + b5E+b6F + b12AB+b13AC + b14AD+b1AE + b16AF+b23BC + b24

BD + b25BE+b26BF + b34CD+b35CE + b36CD+b45DE + b46DF+b56EF (quadratic, 3)

Y = b1A+b2B + b3C+b4D + b5E+b6F + b12AB+b13AC + b14AD+b1AE + b16AF+b23BC+

b24BD+b25BE+b26BF+b34CD+b35CE+b36CD+b45DE+b46DF+b56EF+b123AB+b124ABD +b125ABE+b126ABF+b134ACD+b135ACE+b136ACF+b145ADE+b145ADF

+b156AEF+b234BCD+b235BCE+b236BCF+b245BDE+b245BDF+b245BEF+b345CDE+b346CDF+b356CEF+b456DEF (special qubic, 4)

where A is Semolina, B is soy protein isolate 90%, C is pea protein isolate 80%, D is whey protein isolate 80%, E is oat flour, F is gluten, and b is constant coefficients for linear and nonlinear terms.

## 3 RESULTS AND DISCUSSION

### 3.1 Nutritional composition of the ingredients

According to Table 2, the highest amount of protein was found in SPI and PPI, respectively. Protein had a positive effects on the texture and nutritional values of pasta. SPI, PPI, and WPI proteins were more effective on the nutritional properties. However, G was very effective on texture, due to its network‐forming capacity (Mariotti, Iametti, Cappa, Rasmussen, & Lucisano, 2011). The greatest amount of fiber was in the OF, which increased the nutritional value of pasta (Spiller, 2001).

**TABLE 2:** Nutritional composition of the components

Components (g/100 g)	Moisture content	Ash value	Carbohydrate	Protein	Fiber	Fat
S	14.20 ± 0.05	0.65 ± 0.02	72.83 ± 0.20	12.50 ± 0.10	3.20 ± 0.04	0.50 ± 0.03
OF	11.00 ± 0.01	3.56 ± 0.06	49.20 ± 0.05	13.12 ± 0.08	19.50 ± 0.2	5.80 ± 0.05
SPI	6.42 ± 0.04	5.23 ± 0.30	2.08 ± 0.09	90.90 ± 0.40	1.04 ± 0.1	0.34 ± 0.05
WPI	4.00 ± 0.30	3.04 ± 0.03	2.50 ± 0.07	82.05 ± 0.05	‐	6.50 ± 0.20
PPI	5.50 ± 0.05	4.50 ± 0.06	3.00 ± 0. 50	82.50 ± 0.20	1.00 ± 0.06	6.00 ± 0.40
G	5.88 ± 0.01	0.90 ± 0.04	8.70 ± 0.20	80.00 ± 0.01	0.60 ± 0.05	1.85 ± 0.08

Note

Data are means ± standard of three replicates.

Abbreviations: G, gluten; OF, oat flour; PPI, pea protein isolate 80%; S, Semolina; SPI, soy protein isolate 90%; WPI, whey protein isolate 80%.

### 3.2 Fitting for the model

**FIGURE 1:**
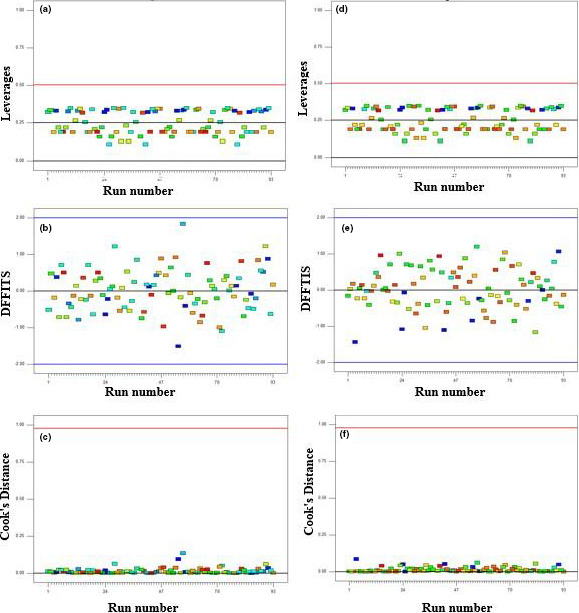
Leverages, difference in fits (DFFITS), and Cook's distance for protein (a, b, and c) and hardness (d, e, and f) models

Linear and quadratic model were selected as the best model for protein and hardness, respectively. The optimal model was selected based on the low standard deviation, low predicted sum of squares, and higher *R*‐squared (Nikzade, Tehrani, & Saadatmand‐Tarzjan, 2012). *p*‐values of the acceptable model were lower than .05, and *p*‐values of lack of fit were higher than .05. Also, adequate precision values of models were higher than 4, indicating the models can be used to navigate the design space (Diedericks & Jideani, 2015). Leverages, difference in fits (DFFITS), and Cook's distance for protein and hardness models are indicted in Figure 1. According to Figure 1 (a and d), all of the leverage values were <0.5, so there was no outliers or unexpected errors in the model. Cook's distance and difference in fits plots also confirmed the validity of the models (Jalali‐Heravi, Parastar, & Ebrahimi‐Najafabadi, 2009).

### 3.3 Effect of enrichment on the protein content of produced samples

According to Table 3, increase in the amount of PPI, WPI, SPI, OF, and G had a significant (*p* < .05) positive effect on the protein's content of the pasta. SPI had the highest effect on the protein content. According to Table 4, the protein range was determined as 27.52–51.80. The highest protein's content obtained with the combination of 36 g S, 12 g SPI, 30 g PPI, 10 g WPI, 7 g OF, and 5 g G (sample 28, Protein content: 51. 80). While the lowest amount of protein was observed in sample 17. The results revealed that the combination of SPI, PPI, WPI, OF, and G increased the protein content by up to 4 times compared to the control sample. Increasing the protein content by using the similar components has been reported previously (Gopalakrishnan et al., 2011; Limroongreungrat & Huang, 2007; Shogren, Hareland, & Wu, 2006). According to data sheet of PPI used, the highest content of amino acids is glutamic acid, aspartic acid, arginine, leucine, and lysine, respectively.

**TABLE 3:** Regression coefficients and correlation for the adjusted model to experimental data in D‐optimal mixtures design for protein and hardness of pasta

AP^*2^	LOF^*1^	R^2^_Pred_	AE	AC	F	E	D	C	B	A	Variables
168.29	0.82	0.99	‐	‐	0.82^a^	0.12^a^	0.77^a^	0.80^a^	0.90^a^	0.12^a^	Protein
45.75	0.38	0.94	−0.20^a^	0.15^a^	9.39^a^	12.94^a^	9.72^a^	2.30^a^	11.51^a^	3.22^a^	Hardness

Note

*1: Lack of fit. *2: adequate precision, and a: Significant at 0.001 level. AB, AD, AF, BC, BD, BE, BF, CD, CE, CF, DE, and DF not significant at 0.05 level. Semolina (A), soy protein isolate 90% (B), pea protein isolate 80% (C), whey protein isolate 80% (D), oat flour (E), and gluten (F).

**TABLE 4:** Hardness and protein of experimental pasta samples

Run	Protein (g/100g)	Hardness (g)
1	31.50 ± 0.40	520.76 ± 3.51
2	35.48 ± 0.05	551.73 ± 3.60
3	48.49 ± 0.25	651.16 ± 3.56
4	47.33 ± 0.08	643.23 ± 4.58
5	27.57 ± 0.25	425.16 ± 4.04
6	46.51 ± 0.03	641.60 ± 2.08
7	40.38 ± 0.20	598.19 ± 2.40
8	48.83 ± 0.24	650.98 ± 2.64
9	51.39 ± 0.50	660.10 ± 1.02
10	35.58 ± 0.38	554.17 ± 6.80
11	34.47 ± 0.40	540.12 ± 5.29
12	44.32 ± 0.15	626.04 ± 2.75
13	35.69 ± 0.06	556.84 ± 1.52
14	30.29 ± 0.10	497.53 ± 1.56
15	43.14 ± 0.07	619.64 ± 2.03
16	27.63 ± 0.41	427.033 ± 2.51
17	27.52 ± 0.05	424.00 ± 3.60
18	38.11 ± 0.10	568.89 ± 2.08
19	34.57 ± 0.15	531.86 ± 4.72
20	50.79 ± 0.30	670.55 ± 3.05
21	34.46 ± 0.43	547.43 ± 3.05
22	42.53 ± 0.21	610.04 ± 0.577
23	44.73 ± 0.36	626.37 ± 5.50
24	33.29 ± 0.10	523.46 ± 5.00
25	39.50 ± 0.37	587.45 ± 6.00
26	39.42 ± 0.23	578.85 ± 1.10
27	43.52 ± 0.41	618.83 ± 3.87
28	51.80 ± 0.30	653.27 ± 5.27
29	47.22 ± 0.05	637.48 ± 5.84
30	48.23 ± 0.29	650.31 ± 7.63
31	34.48 ± 0.41	547.10 ± 3.60
Control	12.58 ± 0.44	551.07 ± 7.21

Note

Data are means ± standard of three replicates.

Filip and Vidrih (2015) reported that the addition of PPI (40%) to pasta increased the protein and essential amino acids content by 4 and 2.3 times compared to the control sample, respectively. Sindayikengera and Xia (2006) indicated that the limiting amino acids in WPI (80%) were valine, phenylalanine, tyrosine, and isoleucine while it was rich in methionine, cysteine, lysine, and threonine. Also, Gopalakrishnan et al. (2011) reported that the lysine content of pasta fortified by WPI was much higher than the requirement for adults and children. Limroongreungrat and Huang (2007) indicated that enrichment pasta by SPI (45%), increased the protein content by 15.9 times compared to pasta made from sweet potato flour. Naseri, Taslimi, Seyedin, Haratian, and Abadi (2009) reported that addition of SPI at 2% to pasta increased the amount of protein and lysin about 13 and 32%, respectively. Due to the high protein content and nutritional value of the amino acids used, this product can be considered suitable for athletes.

### 3.4 Effect of enrichment on the texture of produced samples

As the hardness analysis showed (Table 3), OF, SPI, WPI, and G had a strong effect on the hardness of pasta, respectively. The results showed that interaction of S/PPI and S/OF had a significant effect on the hardness of pasta. Interaction between S and PPI increased the hardness of pasta whereas interaction between S and OF reduced the hardness of samples. However, interaction between S and PPI and S and OF had the lowest effect on the hardness of samples with the coefficients of 0.15 and −0.20, respectively. Interaction of other components was not significant at 0.05 level. As can be seen from Table 4, the sample containing S (36 g), SPI (12 g), PPI (30 g), WPI (10 g), OF (7 g), and G (5 g) (sample 28) showed the highest amount of hardness among the other samples. In this sample, the hardness was 1.18 times higher than hardness of control sample. Increasing the hardness of pasta has been reported by increasing the protein content (Alireza Sadeghi & Bhagya, 2008; Bhatt, Jatav, Kiledar, & Srivastava, 2015; Shyam et al., 2017; Wee, Loud, Tan, & Forde, 2019). Teterycz et al. (2020) reported that by increasing the protein content, the hardness of pasta increased. Wee et al. (2019) reported the hardness of noodles increased by increasing the protein content. Voisey, Wasik, and Loughheed (1978) suggested that hardness depends on the amount of starch and starch gelatinization in the pasta. During cooking by the diffusion of water, a lot of changes occur in the microstructure of pasta such as starch gelatinization. In fact, protein enrichment has led to the strengthening of the protein network which reduces the starch gelatinization. Also, Laleg et al. (2017) suggested by replacing of flour with protein source, the total gluten content was reduced which leading to the higher hardness. In fact by reducing the gluten content, the water absorption decreased (Laleg et al., 2017; Teterycz et al., 2020). In addition, the results indicated by increasing the amount of fiber (oat flour), the hardness of samples increased. Similar results have been reported by the other researchers (Chusak et al., 2020). By increasing the amount of fiber, the hydrophilic properties increased and caused a reduction in the swelling index of pasta (Chusak et al., 2020). Lisiecka, Wójtowicz, Dziki, and Gawlik‐Dziki (2019) reported that the competition between starch, protein, and fiber for water absorption and different hydration levels of the components may affect the strength of the gluten network.

### 3.5 Optimization

Optimization was performed to obtain the optimal amount of each compound with the aim of producing the appropriate pasta for athletes. Our goal was to access the maximum protein content while the hardness was in the recommended range of 550–560 (according to the texture acceptability). The determined optimized points were 45.41%, 5%, 24%, 5%,18%, and 2% for S, SPI, PPI, WPI, OF, and G, respectively. Under the optimum conditions, protein content and hardness were 37.25 g/100 g and 560 g, respectively. The chosen combination attained 0.90 desirability score. Desirability value higher than 0.8 indicates that the quality of product is acceptable and excellent (Amini Sarteshnizi, Hosseini, Bondarianzadeh, & Colmenero, 2015). In optimized formulation, the protein content increased by more than 2.9 times compared with control.

### 3.6 Cooking properties of control and enriched pasta

Cooking properties of pasta have a great impact on the quality of pasta (Jayasena & Nasar‐Abbas, 2012). According to Table 5, the optimal cooking time for the OS was longer than the control. Increasing the OCT of pasta has been reported by adding various protein source such as SPI (Bae & Rhee, 1998), mushroom, and defatted soy flour (Kaur, Sharma, Nagi, & Ranote, 2013). Oh, Seib, Ward, and Deyoe (1985) indicated the linear correlation between the OCT and protein content. Cooking loss is generally used to predict the overall performance of pasta cooking (Jayasena & Nasar‐Abbas, 2012), and it is preferred to be <8% (Teterycz et al., 2020). The cooking loss test results indicated that the cooking loss of the OS was 10% higher than control. Similar trends reported by others (Kaur et al., 2013; Teterycz et al., 2020). Laleg et al. (2017) reported by decreasing the gluten the cooking loss increased. Increasing the cooking loss could be a consequence of the dilution of the gluten network and weakening of its overall structure. Also, the higher amount of fiber in OS compared with the control could lead to the dilution of the gluten network and increased cooking loss. (Teterycz et al., 2020).

**TABLE 5:** Color, cooking, and texture properties of control and enriched pasta

Samples	L*	a*	b*	OCT (min)	Cooking loss (%)	Adhesiveness (g.sec)
Control	72.23 ± 0.10^a^	4.91 ± 0.07^b^	33.42 ± 0.09^a^	10.80 ± 0.20^b^	6.48 ± 0.20^b^	−17.00 ± 4.23^a^
OS	60.16 ± 0.05^b^	11.52 ± 0.09^a^	30.27 ± 0.12^b^	16.18 ± 0.17^a^	7.20 ± 0.05^b^	−11.21 ± 2.12^b^

Note:

Data are means ± standard of three replicates. Values with different lowercase letters in the same column are significantly different (LSD, *p* < .05).

Abbreviations: OCT, optimal cooking time; OS, Optimal sample.

### 3.7 Color analysis of control and enriched pasta

**FIGURE 2:**
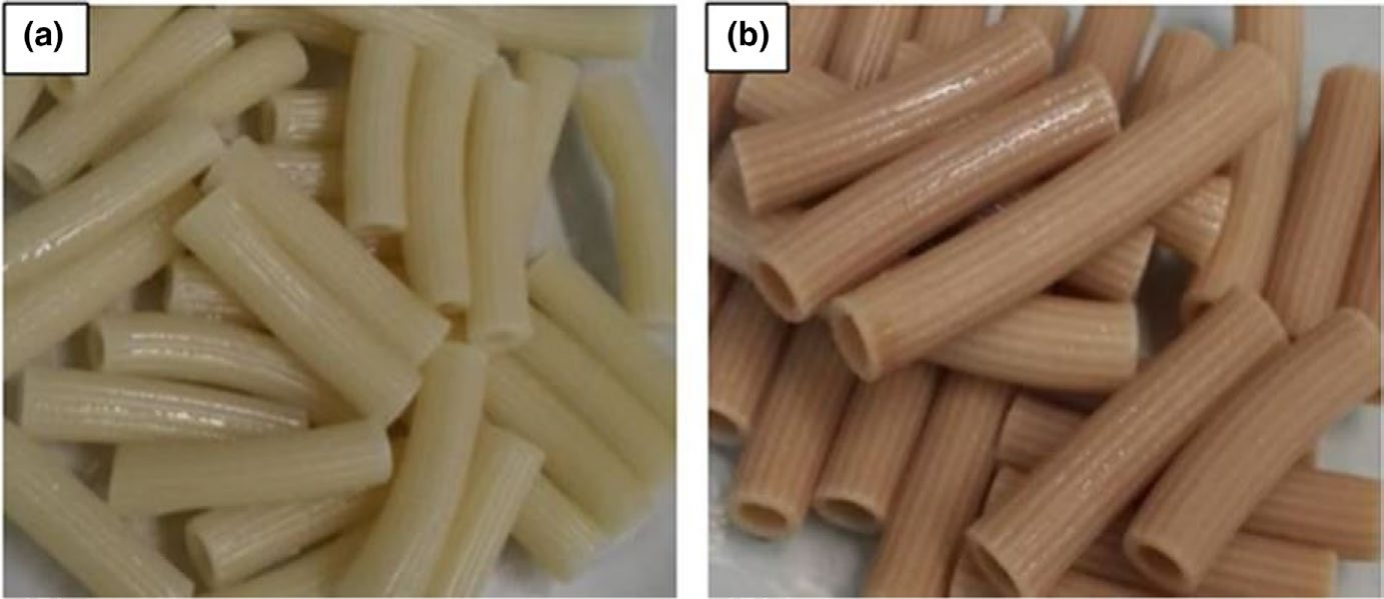
Effect of enrichment on the color of pasta. Control (a) and enriched pasta (b)

The color of pasta was determined in terms of the L^*^, a^*^, and b^*^ values. According to Figure 2 and Table 5, enrichment of pasta formulation affected the color changes of product. The value of L^*^ and b^*^ in control was higher than the OS, while the value of a^*^ was lower than OS (*p* < .05). Our results agree with reports of Alireza Sadeghi and Bhagya (2008), Petitot et al. (2010), and Teterycz et al. (2020). The darker color of OS could be a consequence of the higher amount of ash and color of the added components (Teterycz et al., 2020).

### 3.8 Texture characteristics of control and enriched pasta

Krishnan, Menon, Padmaja, Sajeev, and Moorthy (2012) stated that adhesiveness shows as an evaluation of the stickiness of foods while eating. According to Table 5, the adhesiveness of control sample was higher than the fortified pasta. These results are in agreement with the findings indicated by Alireza Sadeghi and Bhagya (2008) who found that the stickiness of pasta was decreased by increasing amount of mustard protein isolate. Alireza Sadeghi and Bhagya (2008) proposed that the reduction in the stickiness of pasta could be a consequence of the reduction in starch ratio in the enriched pasta or physical entrapment of starch in protein network with increased substitution ratio.

### 3.9 Sensory properties of the cooked control and enriched pasta

Sensory analysis results of uncooked and cooked samples are shown in Table 6. Results of the sensory evaluation of samples on a scale from 1 (dislike extremely) to 9 (like extremely) were indicated that the fortification of pasta significantly reduced the scores of hardness. While the OS had higher scores in terms of overall liking and color compared with the control. Increasing the scores of overall liking could be a consequence of the OS color. Results of color investigation about of color change (instrumental analyses) were confirmed by sensory evaluation results. According to Table 5, there was no significant difference between the control and the OS in terms of flavor. Hanna, Satterlee, and Thayer (1978) and Kaur et al. (2013) reported that the use of different protein sources in pasta formulation has a significant effect on the sensory characteristics of product. Increasing of overall liking in pasta enrichment has been reported with various protein sources such as mushroom powder and Bengal gram flour (Kaur et al., 2013). Despite these results, Shogren et al. (2006) reported that fortification spaghetti with 50% soy flour created beany and bitter flavors compared with control.

**TABLE 6:** Sensory properties of control and enriched pasta

Samples	Flavor	Hardness	Color	Appearance	Overall liking
Uncooked					
Control	‐	‐	7.40 ± 0.09^b^	7.78 ± 0.18^b^	7.48 ± 0.20^b^
OS	‐	‐	8.38 ± 0.12^a^	8.42 ± 0.49^a^	8.20 ± 0.05^a^
Cooked					
Control	8.74 ± 0.33^a^	8.42 ± 0.33^a^	7.98 ± 0.72^b^	7.20 ± 0.11^b^	7.96 ± 0.10^b^
OS	8.63 ± 0.29^a^	7.63 ± 0.12^b^	8.81 ± 0.29^a^	8.32 ± 0.19^a^	8.70 ± 0.36^a^

Note

Data are means ± standard of three replicates. Values with different lowercase letters in the same column are significantly different (LSD, *p* < .05).

Abbreviations: OCT, optimal cooking time; OS, Optimal sample.

## 4 CONCLUSIONS

D‐optimal mixture design was used to optimize the formulation of athletic pasta by SPI, PPI, WPI, OF, and G. Fortification of pasta with SPI, PPI, WPI, OF, and G resulted in a product with a higher protein content and better nutritional characteristics compared to the control sample. In the optimal sample, protein content increased by more than 2.9 times compared with control. By increasing the protein content, the harness, optimal cooking time, and cooking loss of products increased. The results of sensory evaluation showed that changes in color increased the overall liking of enriched treatment compared to the control sample. Based on our results, SPI, PPI, WPI, OF, and G have the potential to be used as an added valuable supplement in the pasta industry to improve the nutritional properties.

## CONFLICT OF INTEREST

There is no conflict of interest in this paper.
